# Acute Heart Failure in an Almost-Centenarian Patient With Symptomatic Severe Aortic Stenosis Treated With Ivabradine

**DOI:** 10.7759/cureus.32142

**Published:** 2022-12-02

**Authors:** Hiroki Sato, Hidekazu Kondo, Takahiro Oniki, Seiki Nobe, Naohiko Takahashi

**Affiliations:** 1 Department of Cardiology and Clinical Examination, Faculty of Medicine, Oita University, Yufu, JPN; 2 Advanced Trauma, Emergency and Critical Care Center, Oita University Hospital, Yufu, JPN; 3 Department of Internal Medicine, Kunisaki City Hospital, Kunisaki, JPN

**Keywords:** negative inotropic effect, beta blockers, ivabradine, acute heart failure, aortic stenosis

## Abstract

We report the case of a 99-year-old woman with acute chest pain caused by myocardial ischemia due to severe aortic stenosis (AS) and severe anemia. Red blood cells were transfused; however, this increased the preload and worsened pulmonary congestion. Treatment with drugs and non-invasive positive pressure ventilation could not sufficiently improve the pulmonary congestion. Ivabradine was administered to control the resting heart rate, following which the pulmonary congestion significantly improved. Ivabradine is a promising drug for acute heart failure of patients with AS by improving cardiac output by prolonging the left ventricular diastolic filling time without a negative inotropic effect.

## Introduction

Aortic stenosis (AS) is a common valvular heart disease. It has a prevalence of approximately 4-5% in persons older than 65 years [1]. Its severity and prevalence increase with age [2]. Surgical aortic valve replacement or transcatheter aortic valve implantation is recommended in patients with symptomatic severe AS when the benefit of the intervention outweighs its risk, especially considering the patient’s age, comorbidities, and other general conditions [3]. Unlike these interventions, medical therapy has not been found to improve the outcome of patients with symptomatic AS [4]. However, some patients with symptomatic AS are not eligible for interventive treatment due to the presence of comorbidities, dementia, frailty, and an anticipated life expectancy of less than one year, and their intervention-related mortality risk is high [5]. These patients are treated with drugs and other non-invasive therapy if they have symptoms of heart failure, with particular care taken to avoid hypotension due to AS-induced hemodynamic instability.

Heart failure in patients with AS is treated with angiotensin-converting enzyme (ACE) inhibitors and diuretics at low doses with gradual dose titration [6]. It is also treated by reducing the left ventricular (LV) afterload [7]. ACE inhibitors improve hemodynamic parameters, augment effort tolerance, and reduce dyspnea in symptomatic patients with severe AS [8]. To avoid low cardiac output due to reduced LV filling, it is recommended that diuretics be started from a low dose [9]. Beta-blockers have been reported to improve hemodynamic parameters, lower heart rate, and increase systolic ejection time [10]. Beta-blockers should be initiated in patients with hemodynamically stable status [4]. The efficacy and safety of these drugs in patients with AS have not been well established because there are few randomized trials of these drugs among patients with AS [11].

Ivabradine, a selective inhibitor of If current in the sinus node, improves the prognosis of patients with chronic heart failure through heart rate reduction without a negative inotropic effect [12]. The ETHIC-AHF trial, a randomized study of 71 patients with acute heart failure with a reduced LV ejection fraction (HFrEF), suggested that the coadministration of ivabradine and beta-blockers is feasible and safe in patients with acute HFrEF [13]. However, the efficacy and safety of ivabradine in patients with acute heart failure are still unclear because of the small sample size of the ETHIC-AHF trial. The SHIFT-AHF trial is an ongoing randomized trial of the efficacy and safety of ivabradine as an add-on to standard therapy in patients with acute heart failure [14]. Ivabradine may be a promising treatment for acute heart failure in patients with symptomatic AS. However, only a few cases of the use of ivabradine for treating acute heart failure in patients with severe AS have been reported [15,16].

Here, we report a case of acute congestive heart failure in a very elderly patient with symptomatic severe AS treated with ivabradine.

## Case presentation

A 99-year-old woman with sudden-onset chest pain was administered to the hospital. She had a past medical history of severe AS, Alzheimer's dementia, and repeated lower gastrointestinal bleeding. She had been admitted to a local clinic one week previously to investigate the cause of bloody stools. At presentation, she was afebrile, and her vital parameters were as follows: blood pressure, 103/49 mmHg; heart rate, 97 beats per minute (bpm), sinus rhythm; respiratory rate: 25 per minute; and oxygen saturation (SpO_2_), 93% on room air. Her body weight was 34 kg and she was 138 cm tall (body mass index 17.9 kg/m^2^). Physical examination revealed a loud systolic murmur and bilateral inspiratory wheezing.

The results of a complete blood count revealed severe anemia (hemoglobin: 4.6 g/dL and hematocrit: 14.1%) and elevated levels of brain natriuretic peptide (BNP) (755.5 pg/mL, normal range: < 18.4 pg/mL), high-sensitive troponin I (1,748.3 pg/mL, normal range: < 30 pg/mL), and creatine kinase-MB (22 IU/L, normal range: < 12 IU/L). Renal function was normal (creatinine: 0.52 mg/dl, estimated glomerular filtration rate (eGFR): 83 ml/min/1.73 m^2^).

**Figure 1:**
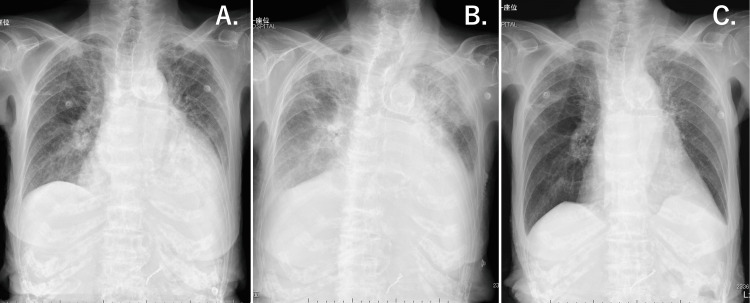
Chest X-ray of the patient (A: day 1, B: day 11, C: day 21)

**Figure 2:**
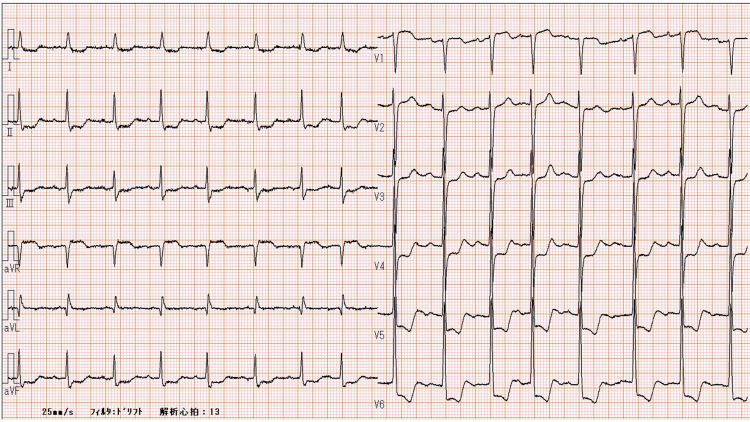
The 12-lead ECG at the presentation (day 1)

Chest radiograph revealed a bilateral consolidation, severe calcification of the thoracic aorta, and an increased cardiothoracic ratio (74%) (Figure 1A). A 12-lead electrocardiogram (ECG) showed ST-segment depression in leads V3-V6, I, II, III, and aVF, slight ST-segment elevation in lead aVR, high voltage of QRS complex as a diagnostic sign of left ventricular hypertrophy, and RR interval alternans associated with pericardial effusion (Figure 2).

**Figure 3:**
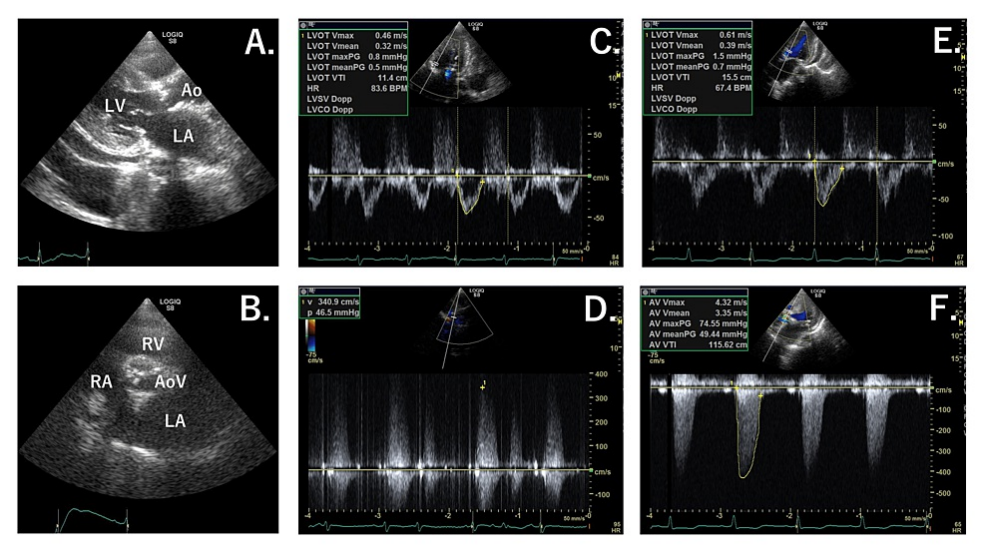
Echocardiography of the patient (A and B: day 1, C and D: day 11, E and F: day 31) Day 1 (A and B): Parameters related to Doppler (LVOT-VTI, AV-VTI) could not be measured because of difficulties by highly calcified aortic valve and patient positioning. Day 11 (C and D): Echocardiography measurements demonstrate a peak transvalvular gradient of 46.5 mmHg and aortic valve velocity of 3.41 m/sec. AVA was 0.37 cm^2^ by planimetry. AV-VTI could not be measured. Day 31 (E and F): Echocardiography measurements demonstrate mean gradient of 49.44 mmHg, maximum gradient of 75 mmHg, mean aortic valve velocity of 3.4 m/sec, and maximum aortic valve velocity of 4.3 m/sec. AVA is 0.39 cm^2^ by planimetry and 0.89 cm^2^ by continuous equation. Her vital parameters at day 31 were as follows: blood pressure, 136/67 mmHg; heart rate, 65 bpm, sinus rhythm. She had no severe anemia (hemoglobin: 10.0 g/dL and hematocrit: 31.8%). LA: left atrium, LV: left ventricle, RA: right atrium, RV: right ventricle, Ao: aorta, AoV: aortic valve, LVOT: left ventricular outflow tract, VTI: velocity time integral, AV: aortic valve, AVA: aortic valve area

Transthoracic echocardiography (TTE) revealed left ventricular wall thickening, a highly calcified trileaflet aortic valve, and a large pericardial effusion with an LV ejection fraction (LVEF) of 62% as measured using the biplane Simpson’s method (Figure 3A, 3B). Inferior vena cava (IVC) collapsed more than 50% on inspiration (expiratory IVC diameter 14.7 mm, inspiratory 3.0 mm). Although AS-related parameters were not measured at presentation, TTE performed two years previously revealed severe AS with an aortic valve area (AVA) of 0.79 cm^_2_^, peak transvalvular gradient of 78 mmHg, and peak aortic valve jet velocity of 4.4 m/sec.

**Figure 4:**
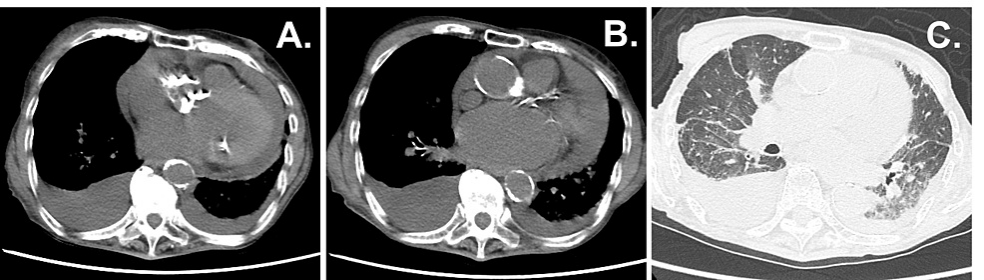
Plain CT of the patient (day 1) Panels A and B show cardiac dilatation, pericardial and bilateral pleural effusion, and severe calcification of the aortic valve, mitral valve, aorta, and coronary arteries. Panel C shows pulmonary congestion.

Plain computed tomography (CT) showed cardiac dilatation, pulmonary congestion, pericardial and bilateral pleural effusion, and severe calcification of the aortic valve, mitral valve, aorta, and coronary arteries (Figure 4).

The patient presented with sudden-onset chest pain. The differential diagnosis included acute cardiac syndromes, aortic dissection, and pulmonary embolism. Laboratory findings such as elevated levels of cardiac enzymes and the ECG and TTE findings suggested the possibility of ischemic heart disease. Acute myocardial infarction could not be ruled out as coronary angiography could not be performed due to the patient’s age and dementia. Aortic dissection and pulmonary embolism were ruled out based on plain and contrast-enhanced CT findings.

**Figure 5:**
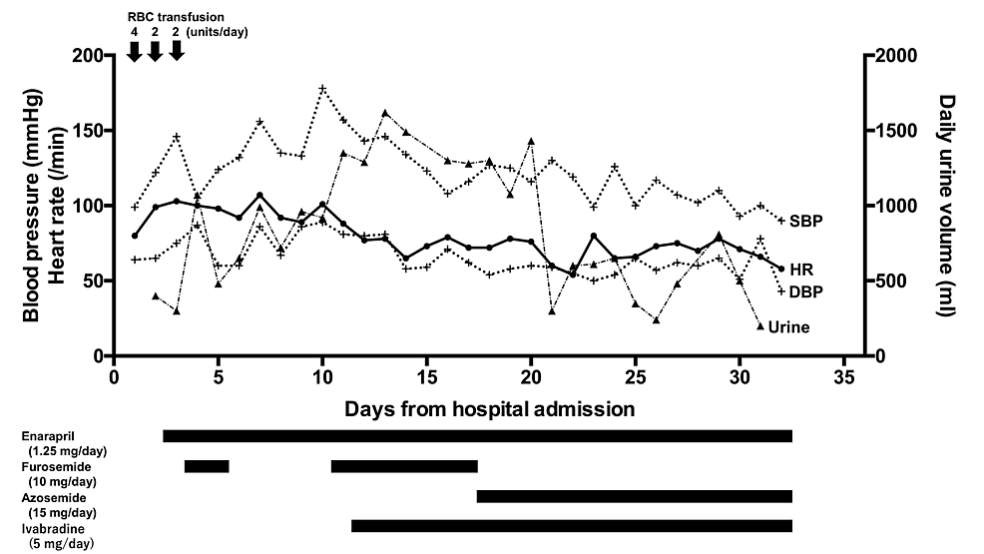
The clinical course of the patient RBC: red blood cell, SBP: systolic blood pressure, DBP: diastolic blood pressure, HR: heart rate, Urine: daily urine volume

The patient was initially diagnosed with myocardial ischemia that was associated with severe AS and aggravated by reduced oxygen supply due to severe anemia. Balloon aortic valvuloplasty for severe AS may be considered as a treatment option, however, her guardian did not want invasive treatment. The clinical course of the patient’s disease is described in Figure 5.

Eight units of packed red blood cells (RBC) were transfused to improve the severe anemia from day 1 to day 3 of admission (day 1: four units, day 2: two units, day 3: two units). Enalapril (1.25 mg/day) was started, and furosemide (10 mg/day) was intravenously administered. The hemoglobin level improved from 4.6 g/dL at presentation (day 1) to 11.8 g/dL on day 4. Pulmonary congestion and pleural effusion worsened after the RBC transfusion. The patient’s weight increased from 34.0 kg (day 1) to 35.4 kg (day 11). She required 3 L of oxygen per minute to maintain a SpO_2_ > 90%. She continued to have tachypnoea (respiratory rate: 25 per minute), sinus tachycardia (heart rate: 101 bpm), high blood pressure (178/89 mmHg), and intermittent chest pain (day 10). Adequate urine output was maintained with intravenous furosemide administration. Non-invasive positive pressure ventilation (NIPPV) was administrated between day 6 to day 9; low-pressure settings were used to prevent excessive reduction of venous return. However, these treatments failed to sufficiently improve pulmonary congestion. The chest radiograph showed a bilateral butterfly shadow (Figure 1B). TTE at day 11 revealed a decreased LVEF (40%). IVC collapsed less than 50% on inspiration (expiratory IVC diameter 16.2 mm, inspiratory 10.8 mm). The AVA by planimetry was 0.37 cm^_2_^, peak pressure gradient was 47 mmHg, and peak aortic jet velocity was 3.4 m/sec (Figure 3C, 3D).

On day 12, ivabradine (2.5 mg, twice a day) was started to control the resting heart rate; the rest of the treatment remained the same. Beta-blockers were not used because of concerns about worsening the hemodynamic status by decreasing cardiac contractility. Subsequently, the resting heart rate dropped from 101 bpm (day 10) to 65 bpm (day 14), and the body weight decreased from 35.4 kg (day 11) to 30.5 kg (day 23). Pulmonary congestion was improved (Figure 1C). The patient’s tachypnea and intermittent chest pain significantly improved. The BNP level slightly decreased from 755.5 pg/mL on day 1 to 539.2 pg/mL on day 17. TTE performed on day 31 revealed an LVEF of 65%, AVA of 0.39 cm^2^ by planimetry and 0.89 cm^2^ by the continuous equation, peak pressure gradient of 75 mmHg, mean pressure gradient of 49 mmHg, peak aortic jet velocity of 4.3 m/sec, and no LV outflow tract (LVOT) obstruction (Figure 3E, 3F).

She was discharged to a nursing home on day 32 of admission. Six weeks after discharge, she was admitted to the hospital due to infective endocarditis. Unfortunately, she did not respond to antibacterial therapy and died three days after admission.

## Discussion

We report a case of severe AS with congestive heart failure after RBC transfusion for severe anemia that responded to treatment with ivabradine, which was started for heart rate control. In patients with severe AS, even if the coronary arteries are normal, supply and demand mismatch during hemodynamic stress may lead to subendocardial ischemia [17]. The patient’s sudden-onset chest pain at presentation could have been due to cardiac ischemia caused by reduced oxygen supply to the myocardial cells resulting from severe-AS-induced decreased coronary perfusion that was aggravated by severe anemia due to lower gastrointestinal bleeding. Treatment of severe anemia with RBC transfusion is generally beneficial for myocardial ischemia; however, in our case, the transfusion-induced increase in preload aggravated LV dysfunction, resulting in significant exacerbation of pulmonary congestion. Pulmonary congestion was significantly improved following the addition of ivabradine to treatment with nasal intermittent positive pressure ventilation (NIPPV), loop diuretics, and an ACE inhibitor.

In patients with severe AS, a decreased AVA limits the aortic flow. An adequate cardiac output is maintained by increasing the heart rate and myocardial contractility through a compensatory increase in sympathetic activity. However, an excessive increase in heart rate can result in a short diastolic period, leading to decreased myocardial perfusion [15]. Inadequate myocardial perfusion decreases myocardial contractility, resulting in decreased cardiac output. Heart rate control can be useful for improving the coronary perfusion and cardiac output of patients with severe AS with tachycardia. In our case, pulmonary congestion resulted in dyspnea, which stimulated the sympathetic nerve system and aggravated tachycardia. Tachycardia-induced decreased cardiac output can further aggravate pulmonary congestion. Ivabradine for heart rate control was suggested to increase cardiac output and improve pulmonary congestion by prolonging the diastolic period. Improvement of anemia would also be related to the reduction of resting heart rate.

Beta-blockers can be useful for heart rate control. Post-hoc analysis of the SEAS (Simvastatin and Ezetimibe in Aortic Stenosis) study, which included 1,873 asymptomatic patients with mild to moderate AS and preserved LVEF who were treated with beta-blockers, revealed significantly decreased hazard ratios of all-cause mortality, cardiovascular death, and sudden cardiac death [18]. However, we did not use beta-blockers for our case due to concerns about worsening the hemodynamic status. Because she still had symptomatic pulmonary congestion. Canine experiments have shown that ivabradine led to significantly greater increases in the diastolic period at both spontaneous and paced heart rates compared with the beta-blocker atenolol [19]. Prolonging the LV diastolic period using ivabradine can improve cardiac perfusion and cardiac output without a negative inotropic effect. Although our case maintained a sinus rhythm during the treatment period, it should be noted that the use of ivabradine increases the risk of atrial fibrillation [20].

There have been two previous reports of patients with severe AS with acute heart failure treated with ivabradine. In the first case, an 86-year-old man with severe AS and a reduced LVEF of 20% showed improvement in heart failure symptoms following treatment with ivabradine [15]. In the second case, an 88-year-old man with severe AS and a reduced LVEF of 35% was admitted to the hospital due to anginal pain and symptoms of heart failure; he showed symptom improvement without any change in the hemodynamic parameters following treatment with ivabradine and a beta-blocker [16]. Ours is the first report on the efficacy and safety of ivabradine for treating congestive heart failure in a very elderly patient with severe AS and a preserved LVEF.

## Conclusions

Interventional treatment is recommended for symptomatic severe AS; however, drug therapy for AS-induced heart failure, cardiac ischemia, and pulmonary congestion is also essential in inoperable patients and patients waiting for interventional treatment. Considering the hemodynamic instability caused by severe AS, ivabradine can be a promising drug for controlling the resting heart rate of patients with symptomatic severe AS without a negative inotropic effect. Further research is required to investigate the usefulness of ivabradine for treating severe AS.

## References

[1] Otto CM, Lind BK, Kitzman DW, Gersh BJ, Siscovick DS (1999). Association of aortic-valve sclerosis with cardiovascular mortality and morbidity in the elderly. N Engl J Med.

[2] Nkomo VT, Gardin JM, Skelton TN, Gottdiener JS, Scott CG, Enriquez-Sarano M (2006). Burden of valvular heart diseases: a population-based study. Lancet.

[3] Iung B, Cachier A, Baron G (2005). Decision-making in elderly patients with severe aortic stenosis: why are so many denied surgery?. Eur Heart J.

[4] Vahanian A, Beyersdorf F, Praz F (2022). 2021 ESC/EACTS guidelines for the management of valvular heart disease. Eur Heart J.

[5] Iung B, Baron G, Butchart EG (2003). A prospective survey of patients with valvular heart disease in Europe: the Euro Heart Survey on valvular heart disease. Eur Heart J.

[6] Otto CM, Cooper S (2022). Medical management of symptomatic aortic stenosis. UpToDate.

[7] Antonini-Canterin F, Huang G, Cervesato E, Faggiano P, Pavan D, Piazza R, Nicolosi GL (2003). Symptomatic aortic stenosis: does systemic hypertension play an additional role?. Hypertension.

[8] Sen J, Chung E, Neil C, Marwick T (2020). Antihypertensive therapies in moderate or severe aortic stenosis: a systematic review and meta-analysis. BMJ Open.

[9] Sawhney N, Hassankhani A, Greenberg BH (2003). Calcific aortic stenosis in the elderly: a brief overview. Am J Geriatr Cardiol.

[10] Hansson NH, Sörensen J, Harms HJ (2017). Metoprolol reduces hemodynamic and metabolic overload in asymptomatic aortic valve stenosis patients: a randomized trial. Circ Cardiovasc Imaging.

[11] Saeed S, Scalise F, Chambers JB, Mancia G (2020). Hypertension in aortic stenosis: a focused review and recommendations for clinical practice. J Hypertens.

[12] Swedberg K, Komajda M, Böhm M (2010). Ivabradine and outcomes in chronic heart failure (SHIFT): a randomised placebo-controlled study. Lancet.

[13] Hidalgo FJ, Anguita M, Castillo JC (2016). Effect of early treatment with ivabradine combined with beta-blockers versus beta-blockers alone in patients hospitalised with heart failure and reduced left ventricular ejection fraction (ETHIC-AHF): a randomised study. Int J Cardiol.

[14] Su Y, Ma T, Wang Z (2020). Efficacy of early initiation of ivabradine treatment in patients with acute heart failure: rationale and design of SHIFT-AHF trial. ESC Heart Fail.

[15] Huang D, Chan PH, Lam CC, Shea PC, Yiu KH, Tse HF, Siu CW (2015). Ivabradine in severe aortic stenosis with poor left ventricular ejection fraction. J Heart Valve Dis.

[16] Nervo E, Menditto E, Taglieri C, Lombardo E, Piccolo S, Feola M (2010). Efficacia antianginosa dell’ivabradina in un grande anziano con stenosi aortica: Analisi dell’effetto sulla portata cardiaca e sui gradienti transvalvolari. G Ital Cardiol.

[17] Ghosh S, Batta A, Sharma YP, Panda P (2021). Very severe aortic stenosis masquerading as acute coronary syndrome. BMJ Case Rep.

[18] Bang CN, Greve AM, Rossebø AB (2017). Antihypertensive treatment with β-blockade in patients with asymptomatic aortic stenosis and association with cardiovascular events. J Am Heart Assoc.

[19] Colin P, Ghaleh B, Monnet X, Su J, Hittinger L, Giudicelli JF, Berdeaux A (2003). Contributions of heart rate and contractility to myocardial oxygen balance during exercise. Am J Physiol Heart Circ Physiol.

[20] Martin RI, Pogoryelova O, Koref MS, Bourke JP, Teare MD, Keavney BD (2014). Atrial fibrillation associated with ivabradine treatment: meta-analysis of randomised controlled trials. Heart.

